# Supplementary material for: TLR4 signaling drives tissue inflammation, Claudin-5 internalization, and vascular barrier breakdown in a mouse model of neonatal meningitis
Source: eLife. 2026 Aug 18;15:RP110458. doi: 10.7554/eLife.110458 (PMC13485302; doi:10.7554/eLife.110458)
Supplement: Supplementary file 2 [file elife-110458-supp1.doc]

**Supplemental File 1. snRNAseq statistics**

Sample	genotype	condition	number	mean number	mean number

(batch)	of nuclei	of transcripts	of genes

per nucleus	per nucleus

PS3 (2)	*Tlr4^MKO^*	infected	8890	1004.26	500.83

PS12 (5)	*Tlr4^MKO^*	infected	9687	928.43	459.64

PS17 (6)	*Tlr4^MKO^*	infected	6225	646.25	349.69

PS14 (5)	*Tlr4^MKO^*	uninfected	9356	894.39	455.57

PS18 (6)	*Tlr4^MKO^*	uninfected	9441	777.10	406.87

PS1 (1)	*Tlr4^ECKO^*	infected	7615	1410.12	665.22

PS11 (5)	*Tlr4^ECKO^*	infected	7687	649.76	364.03

PS15 (6)	*Tlr4^ECKO^*	infected	9142	708.67	426.62

PS9 (4)	*Tlr4^ECKO^*	uninfected	8248	927.29	467.43

PS10 (4)	*Tlr4^ECKO^*	uninfected	7549	814.97	432.43

PS2 (1)	*Tlr4^CKO/-^* (=control)	infected	7964	1969.53	1055.53

PS6 (3)	*Tlr4^CKO/-^* (=control)	infected	8133	986.80	527.68

PS13 (5)	*Tlr4^CKO/-^* (=control)	infected	8458	650.66	359.79

PS4 (2)	*Tlr4^CKO/-^* (=control)	uninfected	10953	880.44	480.63

PS8 (3)	*Tlr4^CKO/-^* (=control)	uninfected	8853	830.12	417.12

Average	8547	935.00	489.60

total	128,201
