# Supplementary material for: TLR4 signaling drives tissue inflammation, Claudin-5 internalization, and vascular barrier breakdown in a mouse model of neonatal meningitis
Source: eLife. 2026 Aug 18;15:RP110458. doi: 10.7554/eLife.110458 (PMC13485302; doi:10.7554/eLife.110458)
Supplement: Supplementary file 3 [file elife-110458-supp2.docx]

**Supplementary File 2. Number of mice and numbers of images per experiment**

Number of mice per genotype for immunostaining analysis

| Group | ASC | CD206 | Casp3 | Cldn5 | ICAM1 | Streptavidin | ZO1 | Iba1 | RFP-*E. coli* | Sun1-GFP | R26-tdT-2A-GFP |
| --- | --- | --- | --- | --- | --- | --- | --- | --- | --- | --- | --- |
| WT, Uninfected | 2 | 2 | 2 | 3 | 2 | 2 | 2 | 2 | 2 | 0 | 0 |
| WT, Infected | 6 | 7 | 6 | 8 | 7 | 5 | 4 | 6 | 6 | 0 | 0 |
| TLR4^VEcko^, Uninfected | 1 | 1 | 2 | 2 | 2 | 1 | 1 | 2 | 2 | 0 | 0 |
| TLR4^VEcko^, Infected | 4 | 4 | 3 | 10 | 6 | 5 | 7 | 9 | 9 | 0 | 0 |
| TLR4^Mcko^, Uninfected | 0 | 0 | 2 | 2 | 1 | 1 | 0 | 0 | 0 | 0 | 0 |
| TLR4^Mcko^, Infected | 4 | 3 | 3 | 6 | 5 | 2 | 2 | 0 | 0 | 0 | 0 |
| Cdh5CreER/+ Control | 0 | 0 | 0 | 0 | 0 | 0 | 0 | 0 | 0 | 3 | 3 |
| Lyz2cre/+ Control | 0 | 0 | 0 | 0 | 0 | 0 | 0 | 0 | 0 | 3 | 3 |

Number of images per genotype for immunostaining analysis

| Group | ASC | CD206 | Casp3 | Cldn5 | ICAM1 | Streptavidin | ZO1 | Iba1 | RFP-*E. coli* | Sun1-GFP | R26-tdT-2A-GFP |
| --- | --- | --- | --- | --- | --- | --- | --- | --- | --- | --- | --- |
| WT, Uninfected | 8 | 8 | 3 | 51 | 18 | 16 | 7 | 4 | 4 | 0 | 0 |
| WT, Infected | 20 | 32 | 22 | 120 | 43 | 34 | 9 | 12 | 12 | 0 | 0 |
| TLR4^VEcko^, Uninfected | 4 | 6 | 4 | 21 | 7 | 7 | 2 | 4 | 4 | 0 | 0 |
| TLR4^VEcko^, Infected | 19 | 11 | 19 | 74 | 21 | 28 | 13 | 18 | 18 | 0 | 0 |
| TLR4^Mcko^, Uninfected | 0 | 0 | 2 | 8 | 2 | 4 | 0 | 0 | 0 | 0 | 0 |
| TLR4^Mcko^, Infected | 8 | 6 | 4 | 37 | 13 | 8 | 6 | 0 | 0 | 0 | 0 |
| Cdh5CreER/+ Control | 0 | 0 | 0 | 0 | 0 | 0 | 0 | 0 | 0 | 30 | 30 |
| Lyz2cre/+ Control | 0 | 0 | 0 | 0 | 0 | 0 | 0 | 0 | 0 | 3 | 3 |
