# Supplementary material for: TLR4 signaling drives tissue inflammation, Claudin-5 internalization, and vascular barrier breakdown in a mouse model of neonatal meningitis
Source: eLife. 2026 Aug 18;15:RP110458. doi: 10.7554/eLife.110458 (PMC13485302; doi:10.7554/eLife.110458)
Supplement: Peer review history

# Editor report: eLife Assessment

**DOI:** 10.7554/eLife.110458.4.sa0

**Authors:** Jalees Rehman

**Affiliations:** University of Illinois Chicago United States

**Keywords:** Convincing, Important

This study demonstrates that toll-like receptor 4 in non-myeloid cells is a central regulator of leptomeningeal inflammation in the context of neonatal *E. coli* meningitis. The data, derived from conditional gene deletions in mice, and from cultured endothelial cells, are **convincing** and support the authors' conclusions. This work is **important** because it advances our understanding of the host cellular and molecular factors underlying meningitis pathogenesis, especially in the context of the vascular barrier breakdown.

# Referee report: Reviewer #1 (Public review)

**DOI:** 10.7554/eLife.110458.4.sa1

[Editors' note: this version has been assessed by the Reviewing Editor without further input from the original reviewers. The authors have adequately addressed how the percentage of fibroblasts was derived.]

Summary:

In this manuscript, Seegren and colleagues demonstrate that in a mouse model of neonatal *E. coli* meningitis, loss of toll-like receptor 4 (TLR4) in VE-cadherin+ endothelial cells and a subset of meningeal fibroblasts leads to a marked decrease in transcriptional dysregulation across multiple leptomeningeal cell types, a decrease in vascular permeability, and a decrease in macrophage abundance. In contrast, loss of macrophage TLR4 had less pronounced effects. Using cultured wildtype and TLR4-knockout endothelial cells, the authors further demonstrate that TLR4 signaling leads to reversible internalization of the tight junction protein claudin-5, establishing a potential mechanism of increased vascular permeability. Authors also show that claudin-5 internalization is independent of NF-κB. Finally, the authors use RNA-sequencing of wildtype and TLR4-knockout endothelial cells to define the TLR4-dependent cell-autonomous transcriptional response to *E. coli*.

Comments on previous version:

The authors have considerably improved and strengthened the work through the addition of new experimental data, new data analyses, and modifications to their interpretation. Notably, the authors used additional Cre-reporter mice to clarify that Cdh5-CreER is active in endothelial cells and some meningeal fibroblasts, and thus revised nomenclature and interpretation to acknowledge that the Tlr4fl/-;Cdh5-CreER cKO (Tlr4-VEKO) is not exclusively endothelial. The authors also demonstrated that Tlr4-VEKO does not affect peripheral *E. coli* burden, but acknowledge that changes to periphery-derived signals (e.g., cytokines) may contribute to observed leptomeningeal phenotypes.

The authors added PCA plots to show similarity in gene expression shifts across biological replicates (mice). This provides support for the claim that Tlr4-VEKO attenuates infection-associated transcriptional changes. With respect to differential expression analysis, I agree with authors that characteristics of individual cells (e.g. heterogeneity) are of interest. I remain concerned, however, that the formal differential analysis strategy appears to consider cells as independent experimental units, which they are not because a single cell cannot be randomly assigned to an experimental group (control or cKO, uninfected or infected). The mouse is the correct experimental unit for a comparison across these groups because it can be randomized. I appreciate that many of the gene expression changes appear consistent across mice (e.g. Figure 1 - Figure supplement 7) and that there are clear infection- and genotype-associated phenotypes in other assays. I would simply caution that the authors' analysis strategy likely leads to a larger number of type I errors (false positives) than is generally accepted; a mixed (hierarchical) model or pseudo-bulk approach would be more appropriate for future studies.

# Referee report: Reviewer #2 (Public review)

**DOI:** 10.7554/eLife.110458.4.sa2

Summary:

The authors use a postnatal mouse model of *E. coli* bacterial meningitis and a mouse brain endothelioma cell line combined with cell type specific gene deletion to study the function of endothelial TLR4, a cell surface receptor that recognizes gram positive bacterial wall components, in the local leptomeningeal (LPM) response with a focus on endothelial barrier breakdown mediated by TLR4. Single cell transcriptional profiling and imaging studies using wholemount preps of the LPM support that LPM endothelial, CD206+ local macrophage and LPM fibroblast and arachnoid barrier cell inflammatory response and is abrogated in endothelial specific KO of TLR4, pointing to a role for endothelial TLR4 in local LPM response. Culture studies using Bend3.1 cells (a mouse brain endothelioma cell line) support a direct role for TLR4 in the bacteria-mediated inflammatory response and in internalization of Cldn5 via the endosomal-lysosomal pathway, resulting in loss of barrier integrity

Strengths:

The local LPM cell response in meningitis and the role of specific LPM cells in inflammation and CNS barrier breakdown has not been extensively studied, despite ample evidence for primary immune response in the meninges in human patients and in animal models. The authors employ a robust, multi-model approach using both in vivo and in vitro models with cell-type specific knockout to study the function of TLR4 in brain endothelial cell response. The authors nicely combine functional barrier assays with IF for junctional localization in their experimental design and they delve into potential mechanisms of Cldn5 internalization using markers of endosomal-lysomal pathway localization. The authors also describe a new type of barrier assay using a streptavidin-coated plates upon which barrier forming cell cultures can be plated, this could be a very useful alternative or complement to other size-selective barrier assays and presumably could work for other barrier forming cell types, like epithelial cells.

Comments on previous version.

In their revision, the authors addressed prior noted weaknesses with new data and analysis. They now show that TLR4-VE-cad cKO mice have a largely similar disease progression as control mice, including increased bacterial burden in the LPM and brain. This underscores that that the reduced vascular leakage and blunted inflammatory response is due to loss of TLR4 response to bacteria on VE-cad recombined cells and not because the mice are protected from meningitis. The authors also performed additional experiments to show that Cldn5 internalization via the endosomal-lysosomal pathway is independent of NFKB signaling. The authors also added in important discussion points about how their results fit into the broader literature on TLR4 in BBB endothelial cell junctional protein localization and prior work on meningitis in global TLR4.

# Referee report: Reviewer #3 (Public review)

**DOI:** 10.7554/eLife.110458.4.sa3

Summary:

This study investigates the molecular underpinnings of immune responses in the leptomeninges in neonatal bacterial meningitis. Bacterial meningitis is a major disease burden, particularly for neonates, and it has previously been noted that the meningeal immune environment in infants is permissive to opportunistic infection (Kim et al., Sci Immunol, 2023). There is less known about the contribution of the stromal compartment to meningeal immune responses. Seegren et al. interrogate the role of leptomeningeal endothelium in host defense in *E. coli* infected neonatal mice using mouse genetic tools to delete the LPS receptor Tlr4 from either endothelial cells/stromal cells (using Cdh5-CreER) or myeloid cells (using LysM-Cre). The authors use snRNAseq, cleared cortical mounts, and in vitro work to define the impact of *E. coli* infection on leptomeningeal endothelial cells. This study uses a range of innovative techniques to probe the role of the stromal compartment in meningitis. With additional experiments to confirm the specificity of their Cre models, this strengthens the interpretation of the study significantly. The only major weakness is the inability to confirm TLR4 knockout in myeloid cells.

Strengths:

This study makes excellent use of cleared cortical mounts to examine the biology of the leptomeninges, in particular, changes to the endothelium, with unprecedented detail. In combination with high-quality sequencing data provide new insights into the impact of meningitis on the leptomeninges. The data presented by the authors is of very high quality.

The authors have also done substantial work to address my two major comments regarding (1) the specificity of their Cre systems and (2) peripheral impacts of the interventions.

(1) The authors identified and acknowledged some impacts in the leptomeningeal stroma (the relatively high level of recombination in ECs vs FBs presumably reflects a single low dose being given, where other groups have done more aggressive tamoxifen regimens that drive recombination in FBs as well). Given the incomplete recombination in the leptomeningeal FBs, I agree with their conclusion that it is probably endothelial driven. Acknowledging the contributions of other myeloid cells with the L. The Cre-NLS experiments with nuclear markers provided excellent data and had beautiful staining.

(2) The authors did not observe differences in bacterial burden in peripheral organs in either CKO model, suggesting that CNS impacts are not downstream of peripheral bacterial control.

Weaknesses:

(1) While the inducible Cre lines used by the authors target both peripheral and CNS tissues, this potential confound is mitigated by the lack of impact on peripheral disease burden.

(2) The authors were not able to confirm TLR4 knockout in myeloid cells, and this caveat is acknowledged. The lack of response in TLR4 VEKO mice strongly suggests successful conditional knockout.

(3) The cell line model (bEnd.3) is a relatively low fidelity model of BBB endothelial cells. The authors acknowledge this, and it is likely that endothelial cell responses to LPS are highly conserved.

(4) It is perhaps not surprising that Tlr4 is required for meningitis responses with *E. coli*. However, it is unclear if these findings can be generalised to other, more common, meningitis infections (streptococcal/pneumococcal).

# Author comment: Author response

**DOI:** 10.7554/eLife.110458.4.sa4

**Authors:** Philip V Seegren, Amir Rattner, Philip M Smallwood, Jeremy Nathans, Yanshu Wang

**Affiliations:** Johns Hopkins University School of Medicine Baltimore United States; Johns Hopkins University School of Medicine Baltimore United States; Howard Hughes Medical Institute, Johns Hopkins University School of Medicine Baltimore United States; Howard Hughes Medical Institute, Johns Hopkins University School of Medicine Baltimore United States; Johns Hopkins University Baltimore United States

The following is the authors’ response to the previous reviews

We are grateful to you and the reviewers for your careful and positive assessment. In response to the comment below from reviewer #2, we have expanded Figure 1- figure supplement 1 to show the results (images plus quantification) of ERG and PU.1 immunostaining together with DAPI staining that underly the calculation of the percent on non-endothelial cells that are recombined by Cdh5-CreER.

However, I found their explanation as to how they arrived at only 18% of the fibroblasts being recombined confusing. Mostly because it looks like there are many GFP+/ERG- cells in panels A & D, these would be the recombined fibroblasts and AB cells. Considering that Cdh5 gene is pretty broadly expressed across LPM, the prediction is the recombination rate would be higher (recognizing mice strains vary and this is inducible Cre).

Ideally, they would perform recombination analysis with a TF expressed by all fibroblasts in combination with Erg (like Foxc1). However, a potentially simpler approach could be to quantify this using DAPI and Erg in existing images, of the total DAPI+, what are GFP+/DAPI+/Erg- (fibroblasts) vs GFP+/ERG+/DAPI+ (endothelial). The figure would be improved by adding DAPI to one set of panels with GFP/ERG, this would show a lot of DAPI+/GFP- cells, encompassing CD206+ cells and non-recombined fibroblasts.

Thank you for overseeing this manuscript.
